# A call to invest in post-TB lung disease to halt TB transmission in communities

**DOI:** 10.5588/ijtldopen.23.0397

**Published:** 2024-02-01

**Authors:** P. M. Mbelele, F. J. Mtei, F. Thobias, K. S. Msaji, L. Løchting, L. M. Subi, D. Bwana, R. M. Kisonga, P. Neema, P. Howlett, M. Drage, S. K. Heysell, S. G. Mpagama

**Affiliations:** ^1^ Kibong’oto Infectious Diseases Hospital, Kilimanjaro, Tanzania;; ^2^ Department of Global Health and Biomedical Sciences, School of Life Sciences and Bioengineering, Nelson Mandela African Institution of Science and Technology, Arusha, Tanzania;; ^3^ Department of Microbiology and Immunology, School of Medicine, Muhimbili University of Health and Allied Sciences, Dar es Salaam, Tanzania;; ^4^ LHL International, Oslo, Norway;; ^5^ MKUTA, Dar es Salaam,; ^6^ Ministry of Health, National Tuberculosis and Leprosy Programme, Dodoma, Tanzania;; ^7^ Imperial College of London, London, UK;; ^8^ Division of Infectious Diseases and International Health, University of Virginia, Charlottesville, VA, USA.

Dear Editor

We undertook an evaluation of former TB patients for pulmonary rehabilitation. Our diagnostic evaluation aimed to implement clinical standards for post-TB lung disease (PTLD) using operational research.^1^ Nightingale et al. also issued a clinical statement, addressing diverse post-TB morbidities such as social, economic, neurological, cardiovascular, PTLD and psychiatric impairments.^2^ In low- and middle-income African countries (such as Sudan, Ethiopia and Kenya), only 4 in 10 clinicians can diagnose PTLD, contributing to an underestimation of its burden.^3^ Hence, the clinical statement offers guidance on PTLD diagnosis, management, research gaps and recurrent TB detection.^2^ Our findings (presented below) suggest that there may be higher-than-expected community transmission of TB infection among susceptible patients. This may be due to damaged lungs from prior TB disease, weakened immune systems from TB sequelae, or malnourishment,^4^ or high rates of recrudescence of inadequately treated TB from patient’s original infection. Although TB treatment success rate for drug-susceptible strains is approximately 85%, the risk of developing recurrent TB, depending on the setting, ranges from 5% to 47%.^5^ Reports from other settings show individuals with recurrent TB experience substantially longer diagnostic delay than those with new TB diagnosis.^6^

One of the innovative approaches to overcome this delay in TB diagnosis was the development of the practical approach to lung health (PAL) guideline in 2008.^7^ This was devised to manage patients with chronic lung disease while expanding TB diagnostics and high-quality TB services. In many high TB burden settings, including Tanzania, PAL has not yet been fully incorporated into the healthcare delivery system and national TB programmes.^8^ Thus, at Kibong’oto Infectious Diseases Hospital (KIDH), a regional referral centre for TB care in Tanzania, we sought to expand PAL and integrate pulmonary rehabilitation for people with PTLD, in partnership with civil society organisations (CSOs) and communities of TB survivors. Individuals self-identified with PTLD at the outpatient department or were actively referred by CSOs. Individuals were included in the study if they had prior treatment of bacteriologically confirmed TB but presented at KIDH with at least one of the following features: cough, dyspnoeic on mild exertion, excessive fatigability impairing with their daily activities and unable to walk unaided.^3^ All individuals presumed with PTLD were assessed for recurrent TB according to the national algorithm prior to enrolment in the pulmonary rehabilitation programme. All individuals provided informed consent as approved by the local ethical clearance (KIDH in Kilimanjaro, Nelson Mandela African Institution of Science and Technology, and Centre for Educational Development in Health Arusha, Health Research Committee; Tanzania; ref: KNCHREC001).

From October 2021 to December 2022, 554 individuals were assessed for recurrent pulmonary TB (PTB). The CSOs contributed 455 (82%) of the evaluated patients. We found an unusually high proportion of cases of undiagnosed and untreated recurrent TB, including multidrug-resistant strains (Table). We found 135 (24%) had recurrent TB, including 88 (65%) identified from CSO referral. Of these, 133 (99%) were molecular- or culture-confirmed as *M. tuberculosis* complex, eight of whom had rifampicin resistance. The Xpert^®^ MTB/RIF (Cepheid, Sunnyvale, CA, USA) quantification cycle (Cq) defining high (<16), medium (16–22), low (23–28) and very low (>28) for positive results in individual’s sputum was respectively 27 (23%), 35 (30%), 32 (28%) and 22 (19%). Individuals with recurrent TB were more often male and younger than those without recurrent TB. Those with recurrent TB had greater pre-existing occupational exposure to mining, and a longer time from the previous TB episode than those without TB. There was no difference in the distribution of comorbidities such as HIV or diabetes mellitus (DM). Classical TB symptoms, including weight loss, night sweat and loss of appetite, were more common in people with recurrent TB, whereas other clinical features, such as cough, shortness of breath, chest pain and wheeze, which may be present in many non-TB causes of lung disease, were more common in those without recurrent TB. An exploratory multivariable logistic regression, including the a priori variables of age, years since previous TB diagnosis, HIV status, BMI category, DM status and mining occupation was performed. Risk factors associated with a higher risk of recurrent TB were more years since the time of diagnosis (odds ratio [OR] 1.09, 95% confidence interval [CI] 1.03–1.15; *P* = 0.005), male sex (OR 2.66, 95% CI 0.89–10.0; *P* = 0.065), previous mining occupation (OR 1.66, 95% CI 0.96–2.97; *P* = 0.077), being underweight (BMI < 18.5 kg/m^2^) (OR 1.90, 95% CI 1.24–2.90) and younger age (OR 0.97, 95% CI 0.95–1.00).

**Table.** Sociodemographic and clinical characteristics of participants.

Variable	No TB (*n* = 419)	TB (*n* = 135)	Total (*n* = 554)	*P* value
	*n* (%)	*n* (%)	*n* (%)	
Age, years, median [IQR]	47.0 [41.0‒53.0]	45.0 [40.0‒51.5]	47.0 [41.0‒53.0]	0.025
Sex				0.013
Female	40 (9.7)	4 (3.0)	44 (8.0)	
Male	374 (90.3)	131 (97.0)	505 (92.0)	
Missing	5	0	5	
Miner status				0.020
Non-miner	141 (33.7)	31 (23.0)	172 (31.0)	
Miner	278 (66.3)	104 (77.0)	382 (69.0)	
HIV status				0.852
Negative	395 (94.5)	127 (94.1)	522 (94.4)	
Positive	23 (5.5)	8 (5.9)	31 (5.6)	
Unknown	1	0	1	
BMI category				0.003
Normal	267 (63.7)	64 (47.4)	331 (59.7)	
Overweight	9 (2.1)	3 (2.2)	12 (2.2)	
Underweight	143 (34.1)	68 (50.4)	211 (38.1)	
Diabetes				0.529
No	411 (98.6)	134 (99.3)	545 (98.7)	
Yes	6 (1.4)	1 (0.7)	7 (1.3)	
Unknown	2	0	2	
Cough				<0.001
No	120 (28.6)	2 (1.5)	122 (22.0)	
Yes	299 (71.4)	133 (98.5)	432 (78.0)	
Shortness of breath				<0.001
No	42 (10.0)	43 (31.9)	85 (15.3)	
Yes	377 (90.0)	92 (68.1)	469 (84.7)	
Chest pain				<0.001
No	75 (17.9)	55 (40.7)	130 (23.5)	
Yes	344 (82.1)	80 (59.3)	424 (76.5)	
Wheeze				<0.001
No	116 (27.7)	89 (65.9)	205 (37.0)	
Yes	303 (72.3)	46 (34.1)	349 (63.0)	
Weight loss				<0.001
No	398 (95.0)	32 (23.7)	430 (77.6)	
Yes	21 (5.0)	103 (76.3)	124 (22.4)	
Night sweats				<0.001
No	411 (98.1)	25 (18.5)	436 (78.7)	
Yes	8 (1.9)	110 (81.5)	118 (21.3)	
Loss of appetite				<0.001
No	374 (89.3)	40 (29.6)	414 (74.7)	
Yes	45 (10.7)	95 (70.4)	140 (25.3)	
Time since TB diagnosis, years, median [IQR]	4.0 [2.0‒4.0]	4.0 [3.0‒5.0]	4.0 [2.0‒4.0]	0.011
Unknown, *n*	18	0	18	
Xpert MTB/Rif result				<0.001
High	0 (0.0)	27 (20.0)	27 (4.9)	
Low	0 (0.0)	32 (23.7)	32 (5.8)	
Medium	0 (0.0)	35 (25.9)	35 (6.3)	
Negative	419 (100.0)	19 (14.1)	438 (79.1)	
Trace	0 (0.0)	22 (16.3)	22 (4.0)	
Type of TB				<0.001
Drug-susceptible TB	0 (0.0)	127 (94.1)	127 (23.0)	
No TB disease	418 (100.0)	0 (0.0)	418 (75.6)	
Rifampicin-resistant TB	0 (0.0)	8 (5.9)	8 (1.4)	
Unknown	1	0	1	
Diagnostic method				
Clinical	0	1 (0.7)	1 (0.7)	
Culture	0	14 (10.4)	14 (10.4)	
Chest-radiography	0	1 (0.7)	1 (0.7)	
Urine lipoarabinomannan	0	3 (2.2)	3 (2.2)	
Xpert MTB/Rif	0	116 (85.9)	116 (85.9)	

The global TB report for 2022 estimates that 40% of people with active TB disease remain undiagnosed in sub-Saharan Africa, including nearly 35% in Tanzania.^9^ Our findings highlight an additional important niche for the identification of undiagnosed TB patients that should be prioritised. We also made several important observations among this group with symptoms of PTLD: 1) non-respiratory classical symptoms of TB were more common among people with recurrent microbiologically confirmed TB;^10^ 2) long time periods from previous TB diagnoses reduced the likelihood of false-positive Xpert results of residual DNA detection, but otherwise dead bacilli;^11,12^ 3) occupational exposure to mining and subsequent silicosis, and conditions such as ongoing malnutrition, likely placed people at increased risk of recrudescence of TB and reinfection with progression to TB disease; 4) CSOs served as crucial outreach and identified high numbers of people with symptoms of PTLD eligible for pulmonary rehabilitation, of which a considerable portion had recurrent TB. Given that mining was the most common occupation in this cohort, our findings may not be generalisable to the broader TB survivor population. However, it also suggests that prioritising repeated screening among high-risk TB survivor groups, such as those with exposure to silica, or chronic undernutrition despite TB treatment,^13^ may be beneficial. This argument is also consistent with other reports,^2,3^ which suggests that scaling up this initiative may increase notification of people with TB and improve the well-being of individuals with chronic respiratory conditions. At a minimum, this operational research demonstrates that a comprehensive approach to lung health increases the number of individuals seeking care in health facilities with access to TB diagnostics and clinical expertise, increasing notifications for TB.

## References

[1] Migliori GB, . Clinical standards for the assessment, management and rehabilitation of post-tuberculosis lung disease. Int J Tuberc Lung Dis 2021;25(10):1–43. 34615577 10.5588/ijtld.21.0425 PMC8504493

[2] Nightingale R, . Post-TB health and wellbeing. Int J Tuberc Lung Dis 2023;27(4):248–283. 37035971 10.5588/ijtld.22.0514 PMC10094053

[3] Binegdie AB, . Post-TB lung disease in three African countries. Int J Tuberc Lung Dis 2022;26(9):891–893. 35996284 10.5588/ijtld.22.0261 PMC9423016

[4] Padmapriyadarsini C, . Undernutrition & tuberculosis in India: situation analysis & the way forward. Indian J Med Res 2016;144:11–20. 27834321 10.4103/0971-5916.193278 PMC5116882

[5] Mirsaeidi M, Sadikot RT. Patients at high risk of tuberculosis recurrence. Int J Mycobacteriol 2018;7(1):1–6. 29516879 10.4103/ijmy.ijmy_164_17

[6] Xie Z, . Factors associated with diagnostic delay in recurrent TB. BMC Public Health 2020;20(1):1–6. 32770986 10.1186/s12889-020-09005-9 PMC7414540

[7] World Health Organization. Practical Approach to Lung Health-Manual on Initiating PAL Implementation. Geneva, Switzerland: WHO, 2008. 26269870

[8] Banda H, . The “practical approach to lung health” in sub-Saharan Africa: a systematic review. Int J Tuberc Lung Dis 2016;20(4):552–559. 26970167 10.5588/ijtld.15.0613 PMC4784471

[9] World Health Organization. Global tuberculosis report, 2022. Geneva, Switzerland: 2022.

[10] Mpagama SG, . The burden and determinants of post-TB lung disease. Int J Tuberc Lung Dis 2021;25(10):846–853. 34615582 10.5588/ijtld.21.0278 PMC8504494

[11] Mbelele PM, . TB or not TB? Definitive determination of species within the M. tuberculosis complex in unprocessed sputum from adults with presumed multidrug‐resistant tuberculosis. Trop Med Int Health 2021;26(9):1057–1067. 34107112 10.1111/tmi.13638 PMC8886495

[12] Mbelele PM, . Use of a molecular bacterial load assay to distinguish between active TB and post-TB lung disease. Int J Tuberc Lung Dis 2022;26(3):276–278. 35197168 10.5588/ijtld.21.0459 PMC8886960

[13] Sinha P, . Food for thought: addressing undernutrition to end tuberculosis. Lancet Infect Dis 2021;21(10):e318–e325. 33770535 10.1016/S1473-3099(20)30792-1 PMC8458477

